# Annotations

**Published:** 1908-09-12

**Authors:** 


### ANNOTATIONS.

### Army and Navy Male Nurses.

The first annual report of the Executive Committee of the Army and Navy Male Nurses' Co-operation has recently been published, and explains the work undertaken in the course of the year. It is pointed out that the existence of the Co-operation has entirely depended on donations and subscriptions, and the small commission of 10 per cent. deducted from the nurses' fees. The expenses of the offices at 47B Welbeck Street and of advertising cannot be met at present out of the commission of 10 per cent. on the earnings of the thirty nurses now enrolled on the register, though they have been kept constantly in work, and the applications for nurses are daily increasing. The generosity of the public is relied upon to help the committee in their endeavour to build up a society which, in a very short time, will be free from all claims on charity. At least 150 nurses must be in constant employment before the office expenses can be met by the percentage taken from the nurses' earnings. Advertising is also essential for the success of the scheme. The system of male nursing in England at this moment certainly leaves much to be desired, but the association is doing much to improve it by keeping together a class of men who have already been trained for a valuable calling. On leaving the Service these men should be able to demand a market price for their skilled labour. Over 150 men, who have worked in the Army hospitals, have applied for employment within the last six months. Many of the applications that have reached the office are from men who are still in the Services, all of excellent character and with full nursing certificates. The sum of £2,000 is needed. The committee place on record their appreciation of the services of Miss McCaul, the hon. secretary, in the cause of the Co-operation. At the annual meeting Sir F. Treves was elected president of the Co-operation; while Sir William Allchin, M.D., is chairman, and Dr. Howard Tooth vice-chairman. The hon. treasurer is Dr. Horton-Smith Hartley.

### Physiology, Heredity, and Psychology.

There is no doubt that the study of heredity will receive considerable furtherance from the address of Dr. J. S. Haldane, F.R.S., before the British Association. It is scarcely yet time to regard it as a branch of science with definite lines of demarcation. It is, in fact, so vast that, as the President pointed out, it can contain within its scope the results obtained from physics, chemistry, and physiology. The discoveries in chemistry and physics of the last few years have led to the hope that the ultimate problem of existence may be solved in terms of physical and chemical formula. It is not really enough to explain the action and interaction of the chemical compounds or elements, and then deduce either that this is life, or that life is little less than a further property of matter, according to the idea which led up to the old Phlogiston theory. The point of view from which life should be studied is purely biological. The existence of life must be taken as an axiom, and the problem for the biologist will be to discover rather the causes and circumstances of death. Mechanical explanations based on purely physico-chemical lines, entirely failed to explain such a fundamental physiological fact as reproduction. Physiology, under the influence of biological methods, will be concerned not so much with the problem of reducing organic to inorganic phenomena, but of raising inorganic to the realm of the organic. It is to be hoped also that the presidential address will direct attention to a department of science which is closely bound up with biology. Psychology, however difficult for exact study, must be taken into consideration when any biological or physiological fact is studied. The exact reaction which takes place during the process of thinking is not known, nor, if it were known, would it adequately explain thought or consciousness. But the influence of this thought is far-reaching. The biological theory of life is concerned with the physiological relations which determine the structure of each part and its maintenance. This is a change of front dictated by common sense. Science must invariably seek for a working hypothesis, and when one ceases to work another must be found to take its place. Nor could anyone have imagined that this mechanical explanation would ever be really adequate. To explain heredity as a function of the animal cell is simply to recall the worn-out theory of vitalism, which was nothing less than a vicious circle.

### Property in Ideas.

In a recently produced play Mr. J. M. Barrie draws with great ability and considerable frankness the character of a fellow-countryman who gets on in life largely by means of exploiting the ideas of others. No doubt, as Huxley said, the truth itself is the main thing, and the share of credit due to each particular agent helping to establish the truth, a matter worthy of very little consideration. It is not everyone, however, who can be expected to live up to this fine, though rather quixotic, ideal. In these days, as was recently pointed out in a paper by Dr. Claye Shaw appearing in this journal, the competition for new and fruitful ideas is particularly fierce. It might be added that it is not always the originator who benefits. Too often the praise for making a new discovery goes almost undivided to the worker who is most prominent and strenuous in connection with later stages of the investigation—the magic of the scientific imagination being still unappreciated (partly because of the *rococo* style of the late Professor Tyndall's expositions of the subject) even among cultured people. Of course, men with the requisite grade of imaginative faculty—something more or less approaching what Pater, appreciating Pascal, called "superb guessing"—may not have executive ability to match; theirs may be, so to say, the temperament without the technique. Still, this deficiency on the practical side by no means follows of necessity, and industry without inspiration is an equipment infinitely commoner, and infinitely less valuable. Pioneers do not often get their due from contemporary judgments, and the same is true of these indispensable workers of the first kind. The ethically minded will see to it that those who set them on the right track are not deprived of their deserts.

